# BRD9 is an essential regulator of glycolysis that creates an epigenetic vulnerability in colon adenocarcinoma

**DOI:** 10.1002/cam4.4954

**Published:** 2022-07-02

**Authors:** Qunshan Zhu, Xiang Gu, Wei Wei, Zheng Wu, Fengqin Gong, Xiaoqiang Dong

**Affiliations:** ^1^ Department of General Surgery The First Affiliated Hospital of Soochow University Suzhou China; ^2^ Department of General Surgery Jiangdu People's Hospital Affiliated to Medical College of Yangzhou University Yangzhou China; ^3^ Department of Radiotherapy Jiangdu People's Hospital Affiliated to Medical College of Yangzhou University Yangzhou China

**Keywords:** BRD9, colon cancer, epigenetic modification, glycolysis, I‐BRD9

## Abstract

**Background:**

The intensive interplay between aberrant epigenetic events and metabolic remodeling represents one of the hallmarks of tumors, including colon cancer. The functions of Bromodomain Containing Protein BRD‐9 in colon cancer remains indefinite. We aimed to identify the biological roles and clinical significance of BRD9 in colon cancer.

**Methods:**

The univariate‐ and multi‐variate Cox regression models were used to screen risk epigenetic regulators. Kaplan–Meier analysis and *Pearson* correlation analysis were used to assess clinical significance of BRD9. CCK‐8 assays, colony formation assay, Transwell, and soft‐agar assay were performed to determine the in vitro roles of BRD9. The oxygen consumption rate (OCR) and extracellular acidification rate (ECAR) of colon cancer cells were evaluated by a Seahorse XF Extracellular Flux Analyzer. In vivo models and RT‐qPCR, western blotting, and Chromatin Immunoprecipitation (ChIP) assay were conducted to explore the functional roles of BRD9 in COAD.

**Results:**

In the study, we detected the expressions of 662 epigenetic regulators in COAD and identified a series of 42 hazard epigenetic factors with *p* < 0.05. Low‐throughput MTT assays highlighted that BRD9 is an essential target, and targeting BRD9 could reduce significant decreases of cell growth. BRD9 overexpression could notably elevate proliferation and migration potentialities, whereas, BRD9 ablation abolished these effects. Mechanistically, functional enrichment analysis indicated the potential associations between BRD9 and glycolysis metabolism. In addition, BRD9 epigenetically coordinates the H3K27ac modifications on the promoter regions of ENO2 and ALDOC, inducing enhanced glycolysis activity. Lastly, I‐BRD9 could significantly suppress the growth of colon cancer cells in vitro and in vivo.

**Conclusions:**

Together, our study revealed previously unidentified roles of BRD9 in colon cancer metabolism and tumor progression, indicating that BRD9 could be a valuable therapeutic target for COAD patients.

## Graphical abstract

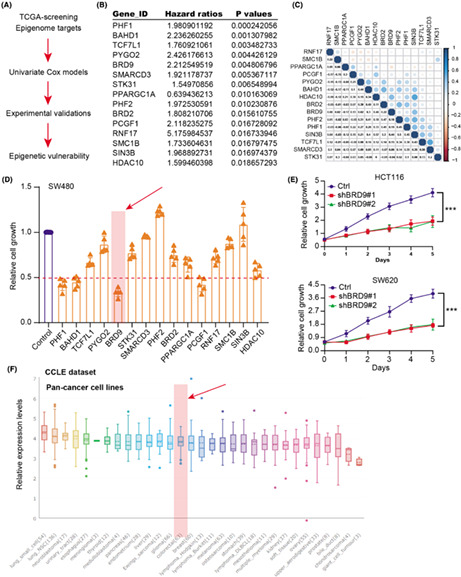

## 1 INTRODUCTION

Colon cancer ranks the third most common malignancy, which is the second cause of cancer‐related death around the world.^1^, ^2^, ^3^ According to the latest statistics in 2021, the estimated new cases of tumors originating from colon are 104,270 of both sexes, and the estimated deaths would come up to 52,980.^4^ Over 90% of all patients, the diagnosed pathological subtype is adenocarcinoma derived from epithelial cells of the colorectal mucosa (COAD).^5^ Currently, the main treatments of COAD patients contain surgical intervention, chemotherapy, as well as radiotherapy.^6^ Besides, targeted drugs (Bevacizumab) or immunotherapies (pembrolizumab) have demonstrated to be effective, however, the overall prognosis of COAD remains to be poor.^7^, ^8^, ^9^ Therefore, intensive efforts are made on the diagnosis and treatment of COAD, including endoscopic diagnosis, tumor markers, as well as molecular targeted therapy. The TNM stages can categorize patients with various prognosis, whereas, there is still a possibility of recurrence in Stage I to III patients who obtained curative resection.^10^, ^11^ The unsatisfactory outcomes mainly due to that most COAD patients being diagnosed at advanced stages are prone to have distant metastases.^11^ The 5‐year overall survival (OS) rates of COAD ranges from 90% of patients with localized disease to 14% in metastatic cases.^12^ As a result, it is of great significance to find more valuable biomarkers for the diagnosis and management in COAD.

Gene mutations or abnormal expressions of hub genes have been known to be important in cancer formation.^13^ In particular, epigenetic alterations have recently been recognized as significant contributors to cancer development, including COAD.^14^, ^15^ Intensive studies have already identified several epigenetic alterations in cancer, including aberrant DNA methylation, histone modifications, ubiquitination, and altered expression levels of different non‐coding RNAs, like long non‐coding RNA(lnc‐RNAs).^16^, ^17^ The previous studies have indicated that abnormal epigenetic events in COAD emerge in early stages and occur more frequently relative to genetic alterations.^18^ Furthermore, advances in genomic technologies have brought out a series of specific epigenetic targets as significant biomarkers or therapeutic vulnerabilities for COAD patients.^19^ Targeting epigenetic alterations in colon cancer not only creates novel biomarker candidates but also facilitates the development of new anti‐tumor therapies. The epigenetic modifying drugs contain inhibitors of enzymes modulating DNA methylation (such as DNMTs and HDACs) and histone modification (such as HMTs and HDMs), some of which have been already validated in clinical trials in COAD.^20^, ^21^ Besides, another application of epigenetic modifiers might be as supplementary drugs for some anti‐cancer therapeutic treatment, including immunotherapy or radiotherapy.^22^ The epigenetic modifiers have been hypothesized to attenuate tumor immune escape and promote the efficacy of immune checkpoint inhibitors, such as PD‐L1. Therefore, a thorough exploration of epigenetic regulatory mechanisms, particularly cancer‐specific epigenetic alterations, would facilitate their clinical use as biomarkers or as therapeutic vulnerabilities in COAD in the future.

As is well documented, the switch/sucrose non‐fermentable (SWI/SNF) complex is a regarded as a common chromatin remodeling context, of which subunits are mutated in various malignancies.^23^, ^24^ It is commonly classified into two subgroups according to the subunit composition and roles, including the barrier‐to autointegration factor (cBAF) complex and polybromo‐associated BAF (PBAF) complex. Moreover, the third subgroup of the SWI/SNF complex was identified in mouse stem cells, which is known as the non‐canonical BAF (ncBAF) complex. The BRD9, glioma tumor suppressor candidate gene 1 (GLTSCR1) and GLTSCR1‐like (GLTSCR1L) are unique constituents of the ncBAF complex. Of note, aberrant BRD9 expressions were frequently reported to be associated with tumorigenesis. The gene copy number of BRD9 frequently occurred in the 5p15.33 region of papillary thyroid carcinoma patients.^25^ BRD9 functions as a critical regulator of androgen receptor (AR) signaling in tumorigenesis of prostate cancer.^26^ Meanwhile, aberrant activation of m6A demethylase FTO could result in BRD9 stabilization, representing a treatment target in HIF2α^low/−^ renal cell carcinoma.^27^ Intriguingly, BRD9 also appears to exert a significant role in tumor suppression. One study found that mutant SF3B1 could recognize an abnormal, deep intronic branchpoint within BRD9 transcripts, which led to the inclusion of a poison exon derived from an endogenous retroviral element and degradation of BRD9 mRNA.^28^ Subsequently, BRD9 ablation induced the loss of ncBAF at the CTCF‐associated loci, thereby promoting melanomagenesis. However, the specific roles of BRD9 in COAD tumorigenesis still remain inconclusive and no relevant researches were currently available.

Here, in the present study, we explored the biological roles of BRD9 in COAD and demonstrated that BRD9 may be a novel predictive biomarker and therapeutic target for COAD progression.

## 2 METHODS

### 2.1 Cell lines and culture

The colon cancer cell lines (HCT‐8, SW620, HCT‐116) and 293 T cells were purchased from America Type Culture Collection (ATCC) and maintained in the Dulbecco's modified Eagle medium (DMEM, #90‐091‐PBR) containing 10% fetal bovine serum (FBS; Gibco, #WS500T), supplemented with 100 U/ml penicillin/streptomycin (ThermoFisher Scientific, #P33067‐100Ml). All cells were maintained in the humidified incubator under the 5% CO_2_ condition at 37°C. According to the manufacturer's instructions, the lipofectamine 3000 (Invitrogen; #L3000008) was utilized to conduct the transfection assays. This published article focused on the epigenetic remodeling and immune regulators.^29^ The list of in vivo epigenetic screen in the study includes epigenetics and some immune regulators. We excluded the immune‐related genes and obtained the final 662 epigenetic regulators in this study. The siRNAs targeting PHF1, BAHD1, TCF7L1, PYGO2, BRD9, STK31, SMARCD3, PHF2, BRD2, PPARGC1A, PCGF1, RNF17, SMC1B, SIN3B, HDAC10, along with negative controls, were purchased from GenePharma.

### 2.2 Samples collection

The fresh COAD samples were collected to detect the mRNA levels of BRD9 in tumor and adjacent normal tissues. The study protocol was approved by the Ethics Committee from the the First Affiliated Hospital of Jiangdu People's Hospital Affiliated to Medical College of Yangzhou University and conducted in accordance with the declaration of Helsinki and German Federal Guidelines. All patients have provided the written informed consent, and the IRB number is 20210039‐R. The age of patients ranged from 47 to 80, with a mean of 60.2 and a median of 59. Total 20 samples of patients with colon cancer were collected in the study from April 2018 to August 2021. All patients with colon cancer were confirmed by pathological diagnosis. All patients did not receive chemotherapy previously at the first diagnosis. The exclusion criteria includes: (1) Patients with advanced clinical stages of tumor or other severe contraindications; (2) patients with previous malignant tumor diseases of other organs; (3) patients with cognitive dysfunction, anxiety, depression, and other psychological disorders; (4) patients with other diseases that impact life quality and physical or mental health. We isolated the total RNA from the fresh COAD tissues using Trizol (#R0016). The 1 μg RNA was reversely transcribed into cDNA using a Superscript First‐Strand cDNA Synthesis Kit (18080‐051; Invitrogen Inc., #11117831001). The RT‐qPCR analysis was conducted via the SYBR Premix Ex Taq II kit (DRR081A; Takara Biotechnology Ltd) according to the manufacturer's descriptions. The relative expression was determined using 2^−ΔΔCt^.

### 2.3 MTT assay

Cells were added in a 96‐well plate at a density of 1 × 10^4^/ml. 20 μl MTT solution (Sigma) was added to each well after incubation for 24, 48, 72, and 96 h. Then, the COAD cells were cultured for 4 h in a humidified incubator. After the supernatants were removed, 200 μl DMSO (#25‐950‐CQC) was added to each well. Finally, the absorbance was detected with a microplate reader (Bio‐Rad).

### 2.4 Stable knockout of BRD9 and overexpression of BRD9

The pX459 was modified to clone guide oligos that target BRD9 gene. Briefly, SW620 or HCT‐8 cells were plated and transfected with pX459 constructs for 24 h, respectively. Then, after 1 day transfection, 1 μg/ml puromycin was utilized to kill cells for 72 h. The living cells were seeded into 96‐well plate to obtain the monoclonal cell line via limited dilution. The BRD9 knockout cell clones are verified by the western blot and validated by sanger sequencing assay. Sequences of gene‐specific sgRNAs are listed as the following: sgBRD9#1:F: 5′‐CACCGCTTCGCCAACTTGTAGTACA‐3′; R: 5′‐AAACTGTACTACAAGTTGGCGAAGC‐3′. sgBRD9#2:F:5′‐CACCGAGGATCAACCGGTTCCTCCC‐3′; R: 5′‐AAACGGGAGGAACCGGTTGATCCT‐3′. For the BRD9 overexpression assays, parental SW620 and HCT‐8 cells were seeded into the six‐well plates with the density of 50,000 cells/well, which were incubated with Lipo2k/plasmid complexes overnight. Followed by PBS washing for three times, the remaining cells were incubated in fresh medium for 2 days. Furthermore, the cells were screened in 5 μg/ml G418 medium after transfection. Lastly, we maintained the cells at 1.5 μg/ml G418 medium after 3 days of culture. In addition to transient infection technology, we also utilized lentivirus technology to generate stable BRD9‐overexpressing cells. Briefly, the overexpression lentivirus system uses the three‐plasmids system containing pLVX‐AcGFP, psPAX2, and pMD2.G. The plasmids were then mixed and transiently transfected into HEK293T cells using the calcium phosphate transfection method. Then, we collected the supernatant. Lentiviruses were purified using the Genecopoeia Lentiviral Particle Purification Kit. After purification, a portion of the virus was collected to detect the virus titer, and the remaining viruses were frozen in a −80°C refrigerator. The selected cells were counted 1 day before virus infection with about 10,000 cells/12‐well plates. After culturing overnight, the virus was diluted with serum‐free medium and added to the 12‐well plate to infect the cells. After 6 h of infection, the virus‐containing solution was removed and we add the medium for cell culture. After 48 h of culture, the fluorescence intensity of cells was observed under a fluorescence microscope. At the same time, puromycin was added to screen the cells for about 2 weeks. We used the western blot to confirm the overexpression efficiency of BRD9 for the following assays.

### 2.5 CCK‐8, colony formation assay, and soft‐agar assay

The cell growth rate was determined via the CCK‐8 assay, colony formation assay and soft agar experiment. First of all, for the CCK‐8 assay, SW620 or HCT‐8 cells were seeded into 96‐well plates (2000 cells/well) and cultured for indicated times. Then, 10 μl CCK‐8 reagent (Abcam, ab228554) was added into the cell culture 4 h before the measurement of absorbance at 450 nm. The colony formation assay was conducted by seeding 500 cells into six‐well plates and we measured the cell colony numbers after 2 weeks. Cells were then fixed with 4% paraformaldehyde (Alpha Diagnoest, USA, #CLM‐229‐PK) and stained with 0.5% crystal violet staining buffer (Sigma‐Aldrich, #SP‐55342‐1). For the soft agar assay, we prepared the sterile 0.5% base agar in the RPMI medium that contains 10% FBS, which was solidified in the six‐well plate. Besides, we also established and mixed the sterile 0.35% top agar with a cell number of 500 per well. When the top agar is solidified, the soft agar plate was maintained under the condition of 37°C. We added the fresh medium into the wells twice every week. Lastly, the cell colonies were fixed via 3.7% paraformaldehyde stained by the 0.01% crystal violet. We finally counted the diameter of the colony which was more than 100 μm under the microscope.

### 2.6 Transwell assay

For migration assay, cells were plated into the apical chamber of 24‐well Boyden plate (8 μM; Corning Glass Works). we placed 4 × 10^4^ COAD cells in 200 μl of serum‐free DMEM in the upper chamber and then added 500 μl of DMEM containing 30% FBS to the lower chamber. For invasion assay, cells were added into the apical chambers precoated with Matrigel. SW620 cells with BRD9 knockout were cultured. Then, the cells were detached with trypsin and transferred to the apical chambers (1000 cells per well) supplemented by FBS‐free DMEM. After 1 day, we counted and compared the cells that were attached to the bottom of the apical chamber.

### 2.7 Western blot

Protein extraction was conducted via the protein extraction kit (Key Gene, KGP9100) from cells. Total proteins were harvested and separated by the sodium dodecyl sulfate polyacrylamide gel electrophoresis (SDS‐PAGE). Then, the nitrocellulose membrane was incubated with antibodies against BRD9 (ab259839, Abcam, 1:1000), against ENO2 (CST#24330), against ALDOC (ab190368, Abcam, 1:1000). β‐actin (ab8226, 1:4000) was selected to be the normalized control. The secondary anti‐body is the Goat Anti‐Mouse IgG H&L (HRP) (ab205719, 1:10,000). The amounts of proteins relative to the loading control were quantified by Image J software. The quantification results of WB bands derived from at least three independent experiments are presented as Means ± SEM in the bar graphs.

### 2.8 Chromatin Immunoprecipitation (ChIP)‐qPCR assay

The parental and BRD9‐KO COAD cells were collected and subjected to ChIP assay. These cells were harvested and sonicated by bioruptor (Diagenode). By using chromatin immunoprecipitation (ChIP) assay kit (Upstate, cat no. 17‐371), the supernatant of sonicated cells was co‐immunoprecipitated with the anti‐BRD9 (Cell signaling Technology, CST#58906), or the anti‐H3K27ac antibody (Abcam, ab4729), or the IgG antibody as the negative control. The enrichment of target gene fragment in DNA precipitate was analyzed by quantitative real‐time PCR. The fold enrichments of target genes between ChIP‐DNA and input‐DNA were determined by ΔCt.

### 2.9 Detection of glucose uptake, lactate production, and ATP levels

The glucose uptake level was detected via the glucose assay kit (Sigma, #CBA086‐1KIT). The lactate level was determined by the Lactate Assay kit (#ARG82235; BioVision). We quantified the ATP level via the CellTiter‐Glo Luminescent Cell Viability Assay (Promega).

### 2.10 Oxygen consumption rate (OCR) and extracellular acidification rate (ECAR)

We seeded the SW620 or HCT‐8 cells into the cell culture plates. We confirmed the eal‐time oxygen consumption rate (OCR) and extracellular acidification rate (ECAR) by the Seahorse Extracellular Flux Analyzer (Seahorse Biosciences). The oxidative phosphorylation and glycolysis of cells from different groups were thus detected and compared. The corresponding assays were conducted as previously described.^30^

### 2.11 In vivo xenograft tumor models and I‐BRD9 treatment

The in vivo assays were reviewed and approved by the Experimental Animal Ethics Committee of Jiangdu People's Hospital Affiliated to Medical College of Yangzhou University (blinded for review). Male BABL/c nude mice (6‐week old) were obtained from the Shanghai SLAC Animal Center (Shanghai, China) and randomly classified into two groups (DMSO group or I‐BRD9 group). Then, 6 × 10^6^ of the SW620 cells were subcutaneously inoculated into the right flank of nude mice. Tumor size was measured every week and tumor volume was calculated as the formula: Length × width^2^ × 1/2. To determine the effects of BRD9 inhibitor (Selleck, #S7835), tumor‐bearing mice were treated by oral gavage with vehicle (10% EtOH, 30% PEG400, 60% MCT [0.5% methyl cellulose, 0.5%Tween 80]) and I‐BRD9 (Tocris Bioscience; 30 mg/kg, dissolved in 10% EtOH, 30%PEG400, 60% MCT), individually. The approved animal study protocal number is YJRY‐2021‐K‐001.

### 2.12 Bioinformatic analysis

The expression levels of BRD9 were obtained from the TCGA‐COAD cohort (https://portal.gdc.cancer.gov/), and limma package was used to determine the BRD9 mRNA levels between tumor and normal samples. The clinical information of COAD patients were shown in Table S1. The Gene Set Enrichment Analysis (GSEA) was conducted using the BRD9 levels (high vs. low) as the phenotype. Based on the GSEA software (gsea‐3.0) downloaded from Broad Institute, we obtained the “c2.cp.kegg.v6.2.symbols.gmt gene sets” from the MSigDB database (http://software.broadinstitute.org/gsea/msigdb) as the reference set. Only gene sets with NOM *p* < 0.05 and FDR *q* < 0.06 were considered as significant. The *survival* package was selected to perform the Kaplan–Meier analysis in R studio (Version 3.6.1). The Gene ontology (GO) analysis was conducted based on the Metascape platform (https://metascape.org/).

### 2.13 Statistical analysis

The statistical analysis was conducted by the GraphPad Prism (V8.0, Prism). Results were indicated by mean ± standard deviation (SD). The differences between groups were determined by Student's *t*‐test or one‐way ANOVA. Overall survival (OS) analysis of patients in indicated groups was conducted by the Kaplan–Meier curves with log‐rank test. The *p* < 0.05 was considered to be statistically significant.

## 3 RESULTS

### 3.1 Epigenome screening and validations highlight that BRD9 is an indispensable epigenetic regulator in colon cancer

**FIGURE 1:**
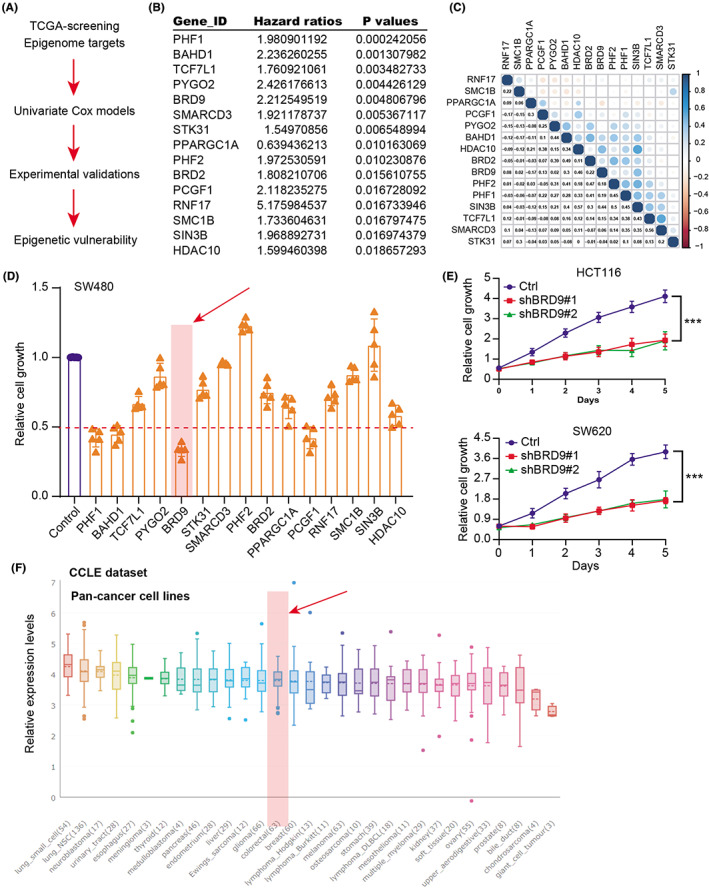
Identification of BRD9 as an essential epigenetic regulator for COAD. (A) Flowchart showing the screen procedure of epigenetic hits. (B) Summary of top 15 candidates with hazard ratios and corresponding *p* values. (C) The correlation heatmap indicating the underlying relationships among indicated regulators. (D) The MTT assays in SW480 cells showing that BRD9 was the most potent target relative to others. (E) CCK‐8 assays indicating the cell growth rates in SW620 or HCT‐116 cells transfected with shCtrl and shBRD9 viruses, respectively. (F) Illustration of BRD9 levels across pan‐cancer cell lines based on the CCLE dataset. Data are displayed as mean ± standard deviation. ***p < 0.001, **p < 0.01, *p < 0.05.

Given that dysregulation of epigenetic regulators contributes to tumorigenesis of COAD, we intended to identify essential chromatin remodeling factors that is indispensable for COAD. We first downloaded the transcriptome data of 662 epigenetic regulators of COAD and carried out univariate Cox analysis to identify a list of 42 hazard epigenetic factors with *p* < 0.05 (Figure 1A and Table S2). The top 15 hits with hazard ratios and corresponding *p* values were summarized in Figure 1B. Besides, the potential relationships across the 15 key epigenetic regulators with prognostic significance were evaluated and illustrated through the correlation heatmap (Figure 1C). Then, we designed specific siRNAs to target these regulators individually, and the knockdown efficiency of each target by siRNA was exhibited in Figure S1A. The in silico validation of MTT assays implicated that BRD9 inhibition induced the most decrease of cell growth relative to other hazard epigenetic regulators (Figure 1D). We also inhibited BRD9 in other two COAD cells (HCT116 and SW620) and confirmed the same results via CCK8 assays (Figure 1E). Lastly, we queried the expression levels of BRD9 in pan‐cancer cell lines via the Cancer Cell Line Encyclopedia (CCLE) dataset (https://sites.broadinstitute.org/ccle) and found BRD9 expresses the middle levels in COAD cells relative to others (Figure 1F). The specific role of BRD9 in COAD still remains unexplored. Therefore, we focused on BRD9 function in COAD.

### 3.2 BRD9 expressed highly in colon cancer that predicts poor prognosis

**FIGURE 2:**
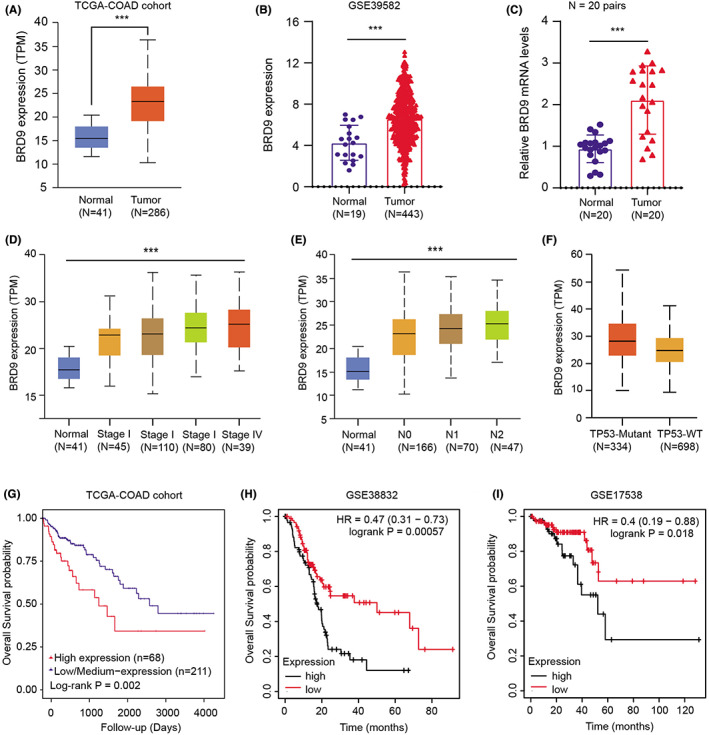
Assessment of clinical significance of BRD9 in colon cancer patients. (A, B) Boxplot exhibiting the differential levels of BRD9 in tumors and normal tissues based on TCGA‐COAD cohort (A) and GSE39582 dataset. (C) The RT‐PCR assay indicated the BRD9 mRNA in the collected COAD tissue samples and normal control. (D–F) Kruskal–Wallis test analysis determing the associations between high BRD9 and hazard clinical characteristics, including pathological stages (D), lymphatic stages (E) and TP53‐mutant features (F). (G–I) Kaplan–Meier analysis with log‐rank test indicated the differential survival outcomes between COAD patients with high BRD9 levels and those with low BRD9 levels based on TCGA‐COAD cohort (G), GSE38832 (H) and GSE17538 (I). Data are displayed as mean ± standard deviation. ***p < 0.001, **p < 0.01, *p < 0.05.

Next, we intended to confirm the expression levels of BRD9 in clinical COAD samples. Firstly, we downloaded the mRNA expression matrix of BRD9 from the TCGA‐COAD cohort, and observed that BRD9 expressed highly in tumor samples versus normal tissues with *p* < 0.001 (Figure 2A). Consistently, we further validated this finding in GSE39582 (*N* = 443) and fresh resected colon cancer samples (*N* = 20 pairs) (Figure 2B,C). Moreover, high BRD9 samples correlated positively with advanced clinical stages and lymphatic stages (Figure 2D,E). Intriguingly, high BRD9 expressions were distributed more in TP53‐mutant samples versus TP53‐WT samples (Figure 2F). Lastly, Kaplan–Meier analysis further showed that patients with high BRD9 expression levels had worse overall survival (OS) outcomes relative to those with low BRD9 levels, which were validated in TCGA‐COAD cohort (Log‐rank *p* = 0.002), GSE38832 (Log‐rank *p* = 0.00057) and GSE17538 (Log‐rank *p* = 0.018) (Figure 2G–I). Taken together, our findings revealed that BRD9 expressed highly in tumor versus normal tissues, which functions as a prognostic factor in COAD.

### 3.3 Oncogenic BRD9 promotes colon cancer proliferation and metastatic abilities

**FIGURE 3:**
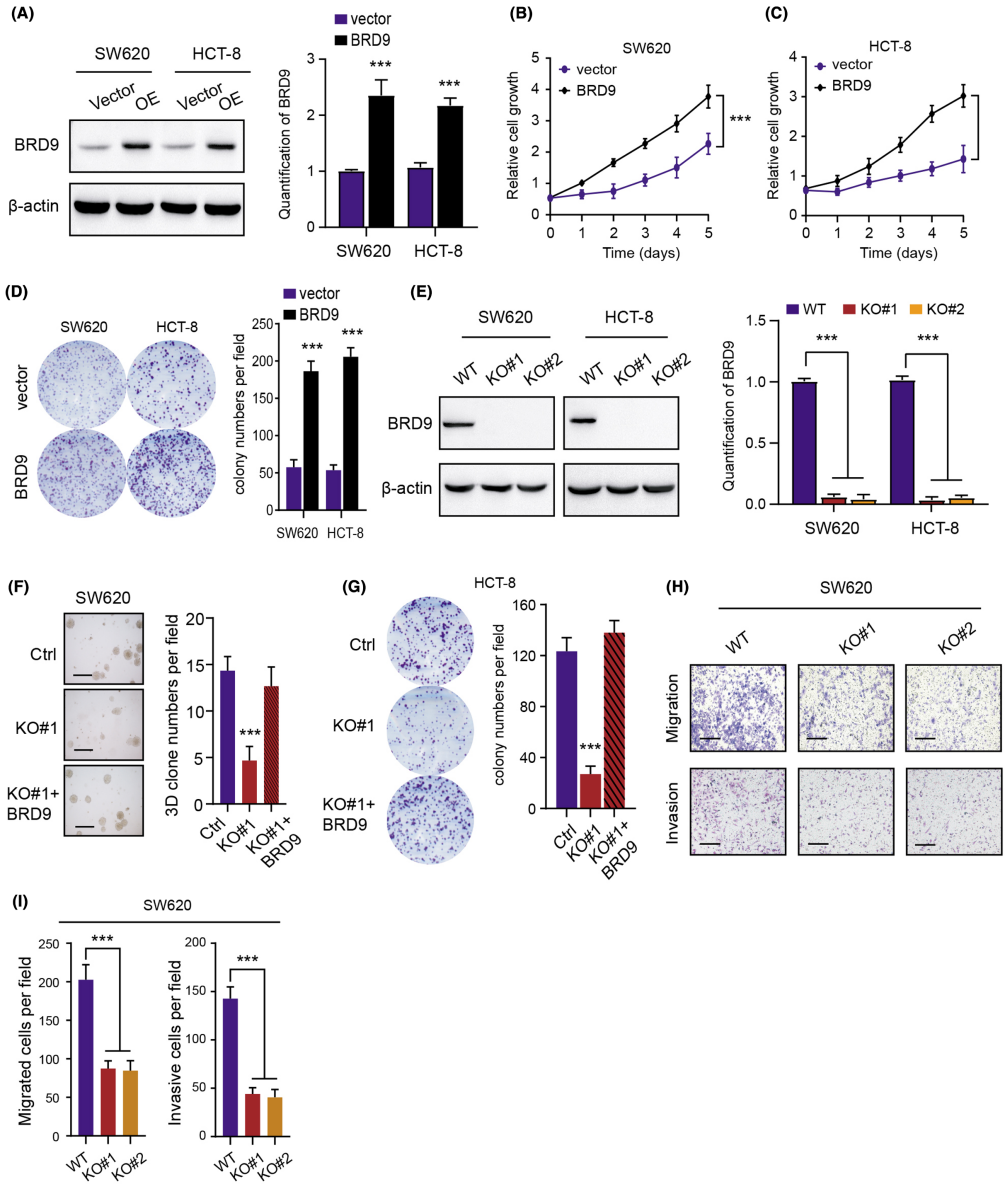
BRD9 functionally promotes cell proliferation and migration of colon cancer. (A) Western‐blotting assay revealing the overexpression of BRD9 in SW620 and HCT‐8 cells relative to control. Quantification of bands with mean ± SD was conducted by ImageJ on the right. (B, C) CCK‐8 assay illustrated the proliferative ability with BRD9 overexpression in SW620 (B) and HCT‐8 (C), respectively. (D) Colony formation assay showed the enhanced ability in BRD9‐overexpressing cells (left panel). Quantification of the results were exhibited and compared on the right panel. (E) Western‐blotting assay revealing the BRD9 deficiency in BRD9‐KO cells versus normal controls in SW620 and HCT‐8 cells. Quantification of bands with mean ± SD was conducted by ImageJ on the right. (F) 3D soft‐agar colony formation assay (left panel) revealing the cell proliferation abilities in three groups (Ctrl; KO#1; KO#1 + BRD9). Quantification of assays were showed on the right statistical panel. Scale bar = 200 μm. (G) Colony formation assay (left panel) revealing the cell proliferation abilities in three HCT‐8 cell subgroups (Ctrl; KO#1; KO#1 + BRD9). Quantification of assays were showed on the right statistical panel. (H, I) Cell migration and invasion assays of SW620 cells were showed after BRD9 was knockout. Representative pictures (scale bars = 200 μm, H) and quantification (I). Data are displayed as mean ± standard deviation. ***p < 0.001, **p < 0.01, *p < 0.05.

To thoroughly understand the underlying mechanisms that BRD9 exerts in COAD tumorigenesis, we firstly utilized the lentivirus infection methods to generate BRD9‐overexpressing COAD cells (SW620 and HCT‐8), which were verified by western blot (Figure 3A). Accordingly, the CCK‐8 assays were performed and we observed that BRD9 overexpression could notably enhance the cell growth ability in SW620 and HCT‐8 cells (Figure 3B,C). Besides, the colony formation potentiality of COAD cells were also markedly enhanced in cells with preferential BRD9 expressions (Figure 3D). Conversely, we also adopted the *CRISPR/Cas9* technology to knock out BRD9 in SW620 and HCT‐8 cells, which were also validated by western blot (Figure 3E). As expected, BRD9 deficiency remarkably suppressed the SW620 cell growth ability in the 3D soft agar, whereas, BRD9 restoration via lentivirus infection technology could completely rescue the impaired colony formation ability (Figure 3F). Consistently, we also found the same results derived from the 2D colony formation assay using the HCT‐8 cells (Figure 3G). Lastly, we also performed the transwell assay and confirmed that BRD9 ablation could significantly suppress the migration ability of SW620 cells (Figure 3H,I). Collectively, our study indicated that BRD9 overexpression could notably enhance cell growth and migration ability, indicating that BRD9 is a robust oncogene in COAD.

### 3.4 BRD9 enhances glycolysis and tumor progression in COAD through activating expression levels of ALDOC and ENO2

**FIGURE 4:**
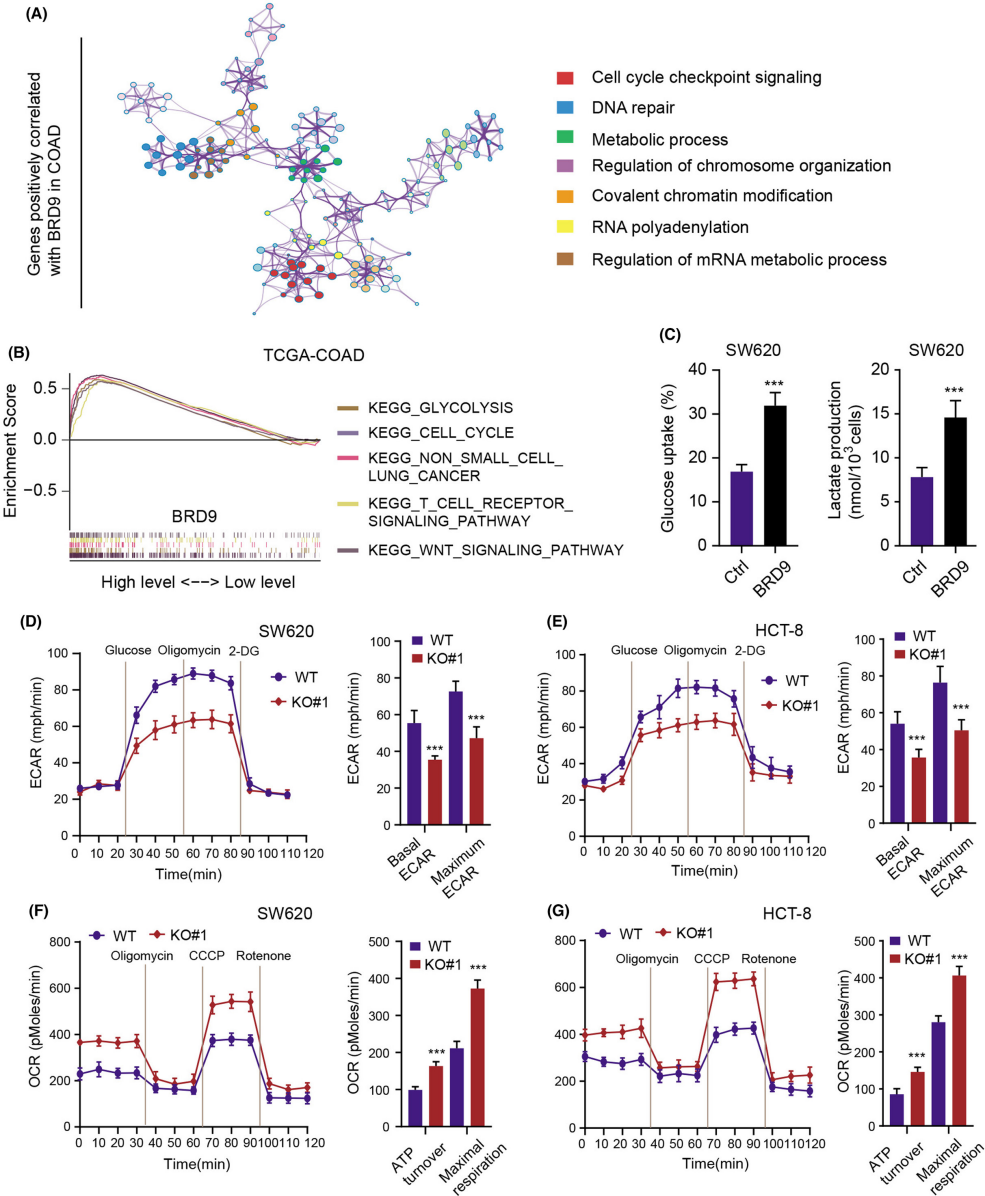
Oncogenic BRD9 regulates the glycolysis activity in colon cancer cells. (A) Gene Ontology analysis (*p* < 0.001 FDR corrected) was conducted and illustrated based on the Metascape platform. (B) GSEA was conducted to identify BRD9‐associated pathways in BRD9‐high versus BRD9‐low samples. (C) Overexpression of BRD9 induced the glucose uptake and lactate production in SW620 cells. (D, E) SW620 or HCT‐8 cells were detected by the Seahorse XP96. Representative profiles of the extracellular acidification rates (ECARs). (F, G) The oxygen consumption rates (OCRs) of SW620 or HCT‐8 cells were exhibited respectively. The following compounds were added into the medium as indicated: Glucose, oligomycin, carbonyl cyanide‐4‐(trifluoromethoxy) phenylhydrazone (CCCP), Rotenone, 2‐deoxy glucose (2DG). Data are displayed as mean ± standard deviation. ***p < 0.001, **p < 0.01, *p < 0.05.

To further figure out how BRD9 regulates colon cancer development, we calculated the BRD9‐associated genes with the correlation coefficient >0.35 (Table S3). Then, 463 genes were selected to perform the Gene Ontology (GO) analysis based on the Metascape platform (https://metascape.org/gp/index.html#/main/step1). We found the significantly enriched biological items based on these correlated genes, including cell cycle checkpoint signaling, DNA repair, metabolic process and regulation of chromosome organization (Figure 4A). Besides, we also catagorized the TCGA‐COAD samples into BRD9‐high and BRD9‐low groups using the median data as the cutoff and conducted the GSEA. We found that several oncogenic crosstalk were notably enriched, like cell clycle, T cell signaling, Wnt signaling and the top hit is the glycolytic metabolism (Figure 4B). As a result, we focused on the regulations between BRD9 and glycolysis in COAD. As shown in Figure 4C, up‐regulation of BRD9 in SW620 cells could notably enhance glucose uptake and lactate production. In contrast, BRD9 loss could suppress glucose uptake and lactate production (Figure S1B). Furthermore, we adopted the Seahorse methodology to detect the glycolysis by the extracellular acidification rate (ECAR) and the mitochondrial oxidative phosphorylation activity using the oxygen consumption rate (OCR) in SW620 and HCT‐8 cells. Expectedly, knockout of BRD9 or I‐BRD9 treatment significantly inhibited glycolytic activities of cells, as indicated by decreased basal ECAR and maximal ECAR in colon cancer cells (Figure 4D,E, Figure S1C). Conversely, BRD9 deficieny or I‐BRD9 treatment notably promoted both basal and maximal OCR in SW620 and HCT‐8 cells (Figure 4F,G, Figure S1C).

**FIGURE 5:**
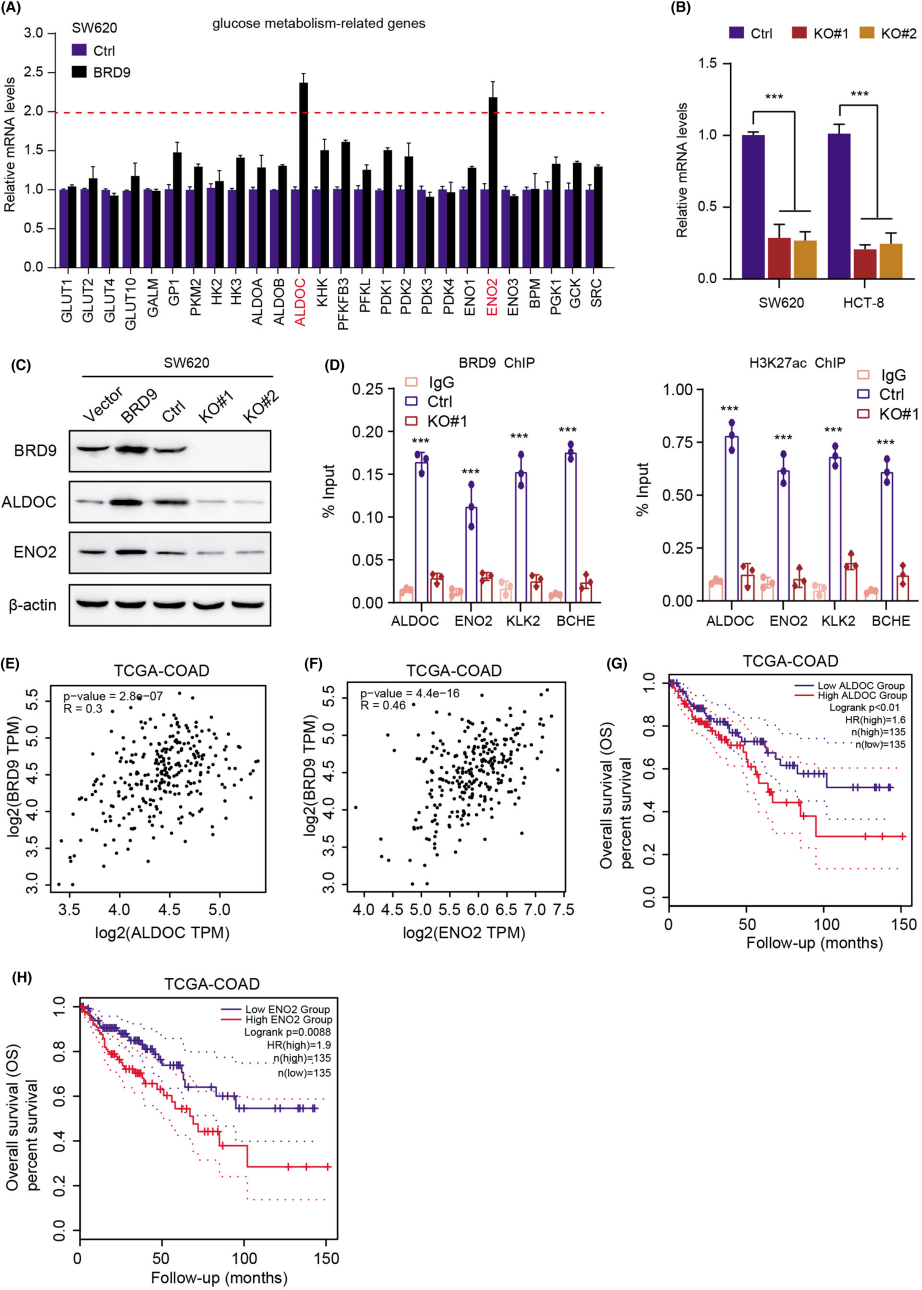
BRD9 enhances glycolysis by targeting ENO2 and ALDOC in colon cancer cells. (A) The ALDOC and ENO2 were identified as BRD9‐regulated genes. The expression of a panel of glucose metabolism‐related genes was assessed by qRT‐PCR in BRD9‐overexpressing SW620 cells. (B) qRT‐PCR assays showing the mRNA levels of ENO2 and ALDOC in BRD9‐deficient and WT cells. (C) Western‐blotting assays detecting the protein levels of ENO2 and ALDOC in the indicated cell samples. (D) ChIP‐qPCR assay of BRD9 binding and H3K27ac in the indicated genes, where KLK2 and BCHE were selected as the positive control. (E, F) Correlation analysis in the TCGA‐COAD cohort revealing the associations between BRD9 and ENO2 or ALDOC, respectively. (G) Kaplan–Meier analysis exhibiting the survival time of patients with high ALDOC and low ALDOC, individually. (H) Kaplan–Meier curve with log‐rank test showing the clinical significance of ENO2 in COAD samples. Data are displayed as mean ± standard deviation. ***p < 0.001, **p < 0.01, *p < 0.05.

Given that BRD9 could manipulate glycolytic pathway, we intended to further elucidate the downstream targets and mechanisms that link BRD9 and glycolysis. Thus, the transcription levels of a panel of glucose metabolism‐related genes were determined in the BRD9‐overexpressing SW620 cells. Intriguingly, the expression levels of only ALDOC and ENO2 were primarily enhanced (fold change >2) with BRD9 overexpression (Figure 5A). In contrast, BRD9 deficieny also reduced the corresponding ALDOC and ENO2 mRNA levels in BRD9‐deficient SW620 cells (Figure 5B). However, ectopic expression of BRD9 could completely restore the ALDOC and ENO2 levels in BRD9‐deficient cells (Figure S1D). Consistently, western blotting assay in SW620 cells also indicated that BRD9 overexpression could elevate the protein levels of ALDOC and ENO2, whereas BRD9 deficiency significantly decreased their protein levels (Figure 5C and Figure S1E). Furthermore, targeting ALDOC or ENO2 by specific shRNAs could notably suppress cell growth of BRD9‐overexpressing cells (Figure S1F). Independent ChIP‐qPCR experiments further validated that BRD9 was present at these gene loci, consistent with a direct role for this subunit in SWI/SNF targeting (Figure 5D). BRD9 could cooperate with H3K27ac to bind at ALDOC and ENO2 promoter to drive their transcriptions. In addition, BRD9 correlated positively with ALDOC and ENO2 expression levels in TCGA‐COAD samples (Figure 5E,F). Lastly, Kaplan–Meier analysis further demonstrated that patients with high ALDOC or ENO2 levels could suffer from worse overall (OS) survival outcomes, compared with those with low ALDOC or ENO2 levels, respectively (Figure 5G,H). Taken together, these data revealed that BRD9 activates transcriptions of ALDOC and ENO2 to enhance glycolytic metabolism and tumor progression in COAD.

### 3.5 I‐Brd9, one BRD9 inhibitor, has clinical significance to suppress the progression of COAD

**FIGURE 6:**
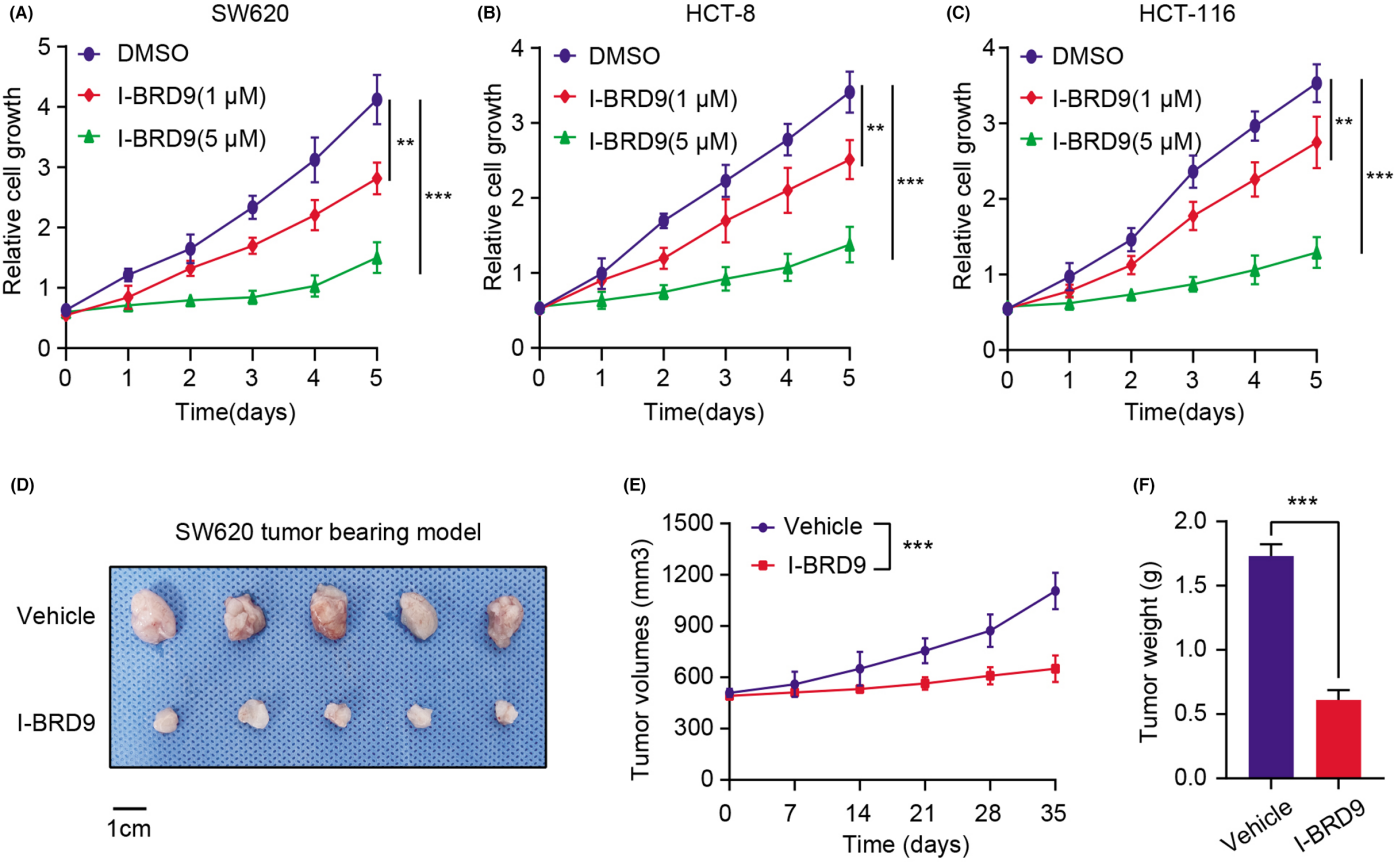
I‐BRD9 was effective to suppress COAD growth in vitro and in vivo. (A–C) CCK‐8 assays showing the impaired cell growth rates of SW620 (A), HCT‐8 (B) and HCT‐116 (C) cells with different doses of I‐BRD9 compound. (D–F) Representative graphs of xenograft colon tumor tissues from the Vehicle and I‐BRD9 groups at Day35 were shown (D), along with quantified tumor volumes (E) and tumor weights (F).

Given the functional importance of BRD9 in regulating glycolysis and progression of COAD, we therefore wanted to examine its potential as a druggable target. As reported, I‐BRD9 was identified through structure‐based design, leading to greater than 700‐fold selectivity over the BET family and 200‐fold over the highly homologous bromodomain‐containing protein 7 (BRD7). We firstly assessed the inhibitory effect of I‐BRD9 in COAD cells and found that I‐BRD9 could suppress in vitro cell growth (SW620, HCT‐8, and HCT‐116) in a dose‐dependent manner (Figure 6A–C). Besides, we also conducted the tumor xenograft studies based on the BALB/c nude mice. We found that daily oral gavage with I‐BRD9 (30 mg/kg per day) indeed arrested tumor growth and even led to regression in xenografts derived from SW620 cells relative to those treated with vehicle, as quantified by tumor sizes and tumor weight (Figure 6D–F). Moreover, in line with the previous results, I‐BRD9 could also reduce the expressions of ALDOC and ENO2 in tumors relative to those derived from the control group (Figure S1G). In conclusion, we proposed that BRD9 represents a druggable vulnerability in colon cancer and I‐BRD9 could be effective to suppress COAD progression.

## 4 DISCUSSION

Epigenetic alterations, like abnormal gene expressions or amplification, could result in the pathogenesis and molecular heterogeneity of malignancies, which has represented the molecular hallmark in tumor.^31^, ^32^, ^33^ Assessment of the “epigenome” in colon cancer has proposed that virtually all colon tumors have aberrantly methylated genes, altered miRNA expression or other abnormal histone modifications.^34^ Besides, the advances in our understanding of epigenetic events in COAD have been systematically developed to be clinical biomarkers for the predictive or therapeutical applications.^1^, ^35^ Progress in this field indicates that these epigenetic alterations could be commonly applied to direct the prevention and treatment of colon cancer.^36^, ^37^ As a result, we intended to screen the potential epigenetic regulators associated with COAD risks and performed bioinformatic analysis based on the large patient samples. Low‐throughout MTT assays revealed that BRD9 inhibition reduced the sharpest decrease of cell growth relative to other regulators. BRD9 expressed highly in tumor samples versus normal adjacent tissues and patients with high BRD9 suffered from worse survival outcomes compared with those with low BRD9. Functional assays further indicated that BRD9 overexpression could markedly increase cell proliferation and migration capacities. Mechanistically, we obseved that BRD9 may have the potentiality to elevate glycolysis, whereas BRD9 deficiency impaired the ability. After detecting a panel of glycolysis‐related signature, we observed that BRD9 could cooperate with H3K27ac to activate the transcriptions of ENO2 and ALDOC. High levels of ENO2 or ALDOC could all lead to poor OS outcomes of COAD patients. Based on these findings, we tested the clinical efficacy of I‐BRD9 and found that I‐BRD9 could significantly suppress the growth of colon cancer cells in vitro and in vivo.

Altered energy metabolism has represented one of the “hallmarks of cancer”, which is the biochemical fingerprint of cancer cells.^38^ The metabolic feature is manifested by preferential dependence on glycolysis to product energy depending on the oxygen‐independent manner.^39^, ^40^ Despite of the fact that glycolysis is less efficient in creating adenosine triphosphate (ATP) versus oxidative phosphorylation, tumor cells have already adapted this metabolism by elevation of glucose up‐take, which in turn enhances glycolysis activity. One study revealed that IGF2BP1, an RNA‐binding protein, was significantly upregulated in colon cells and enhanced stability of LDHA mRNA, thereby driving the glycolysis pathway.^41^ Besdies, FSTL3‐mediated β‐Catenin pathway activation was found to promote EMT and aerobic glycolysis and therefore elevate the invasive and metastatic capacity of CRC cells.^42^ Meanwhile, the metabolic intermediates of glycolysis could also participate in the macromolecular biosynthesis, apart from supplying the cellular energy. The glycolysis remains the predominant energy under hypoxia, which creates the vulnerability that could sensitize cancer cells to inhibition of glycolysis. Collectively, the oncogenic manipulation of glycolysis and multifaceted aspects of glycolytic components underscore the biological significance of tumor glycolysis. Therefore, targeting glycolysis remains meaningful for therapeutic intervention. In this study, we found that BRD9‐driven ENO2 activation results in poor prognosis of patients. Previous studies found that enolase 2 (ENO2) is an essential glycolytic enzyme in the metabolic process of glycolysis, whereas its roles in colon cancer are still unclear. The insulin‐like growth factor‐1 (IGF‐1) was observed to suppress K394 acetylation and promote ENO2 activity in a dose‐ and time‐dependent manner, indicating that inhibition of IGF‐1‐induced ENO2 deacetylation could be a promising strategy.^43^ Calder W Reinsborough et al. also indicated that BCDIN3D regulates ALDOC via a non‐canonical mechanism involving the crucial let‐7 microRNA family and its target site on the 3′UTR of ALDOC.^44^ In line with these knowledge, we also represented that ALDOC is also a therapeutic target associated with glycolysis in colon cancer.

Recently, the BET family has been systematically explored as the treatment targets, and relevant inhibitors as antitumor agents have demonstrated remarkable clinical inhibitory effect, like JQ1 targeting BRD4 in prostate cancer.^45^, ^46^ The Bromodomain inhibitors could be catagorized into two subgroups, including non‐acetylated and acetylated lysine mimetics. The former has relatively weak effects, however the latter directly imitates the binding of an acetylated lysine to the bromodomain and could remarkably preclude the binding of acetylated lysine residues to the hydrophobic binding pocket of the bromodomain. Therefore, given that the biological roles of BRD9 become clear in tumor progression, targeting the bromodomain of BRD9 could provide a novel and attractive tumor treatment selection. For instance, small‐molecule inhibitors of the BRD9 bromodomain effectively attenuate tumor cell proliferation and survival and induce apoptosis. Actually, researchers have already developed multiple effective BRD9 bromodomain inhibitors, including BRD9 selective inhibitors (I‐BRD9,104 BI7273,105 and BI‐9564106) and BRD7/9 inhibitors.^47^ Previous studies have observed that BRD9 binds acetylated K515 on RAD54 and facilitates RAD54's interaction with RAD51, which is essential for Homologous recombination (HR). I‐BRD9, thus acts synergistically with olaparib in HR‐proficient cancer cells.^48^ In line with previous studies, we proposed that I‐BRD9 could also effective in suppressing COAD tumorigenesis.

Nonetheless, there are still several shortcomings in the current study. First of all, we are still uncertain about the specific mechanisms that contribute to high BRD9 in colon cancer. Secondly, large COAD samples derived from multiple cohorts were needed to be collected to further demonstrate the predictive efficiency of BRD9 in COAD prognosis. Thirdly, apart from glycolysis, whether BRD9 regulates other oncogenic crosstalk, like DNA repair, cell cycle or immune responses, remains to be systematically elucidated. Last but not least, the clinical and translational significance of BRD9 in colon cancer should be determined in more abundant in vivo assays, including patient‐derived tumor xenograft (PDX) and orthotopic colon cancer models.

## 5 CONCLUSION

In summary, we conducted the epigenome screens to identify BRD9 as a vital regulator in COAD. BRD9 expressed highly in tumors that correlated with poor prognosis. High BRD9 promotes the proliferation and migration of tumor cells. Particularly, we demonstrated that BRD9 mediates the enrichment of H3K27ac at ENO2 and ALDOC gene loci, thereby enhancing the glycolytic activities of colon cancer cells. I‐BRD9 inhibitor selectively targeting BRD9 could significantly suppress colon cancer cells in vitro and in vivo, representing a therapeutic vulnerability.

## AUTHOR CONTRIBUTIONS

Xiaoqiang Dong conceived the concept of this study. Qunshan Zhu and Xiang Gu conducted the in vitro and in vivo assays. Wei Wei, Zheng Wu, and Fengqin Gong collected the samples from patients. Qunshan Zhu, Xiang Gu, and Wei Wei performed the statistical analysis. Qunshan Zhu wrote the paper and Xiaoqiang Dong conducted the revisions. All authors have approved the final draft of paper.

## CONFLICT OF INTEREST

The authors declare that the research was conducted in the absence of any commercial or financial relationships that could be construed as a potential conflict of interest.

## ETHICAL APPROVAL STATEMENT

The human colon cancer tissue specimens and clinical data was reviewed and approved by Jiangdu People's Hospital Affiliated to Medical College of Yangzhou University (Jiangsu, China). All patients have signed the written informed consent permitted by the Ethics Review Committee of the First Affiliated Hospital of Jiangdu People's Hospital Affiliated to Medical College of Yangzhou University.

## Supporting information


Figure S1

Table S1

Table S2

Table S3

## Data Availability

The molecular experiment data generated and analyzed during the current study are available from the corresponding author on reasonable request.
