# Supplementary figures and images for: BRD9 is an essential regulator of glycolysis that creates an epigenetic vulnerability in colon adenocarcinoma
Source: Cancer Med. 2022 Jul 2;12(2):1572–87. doi: 10.1002/cam4.4954 (PMC9883419; doi:10.1002/cam4.4954)

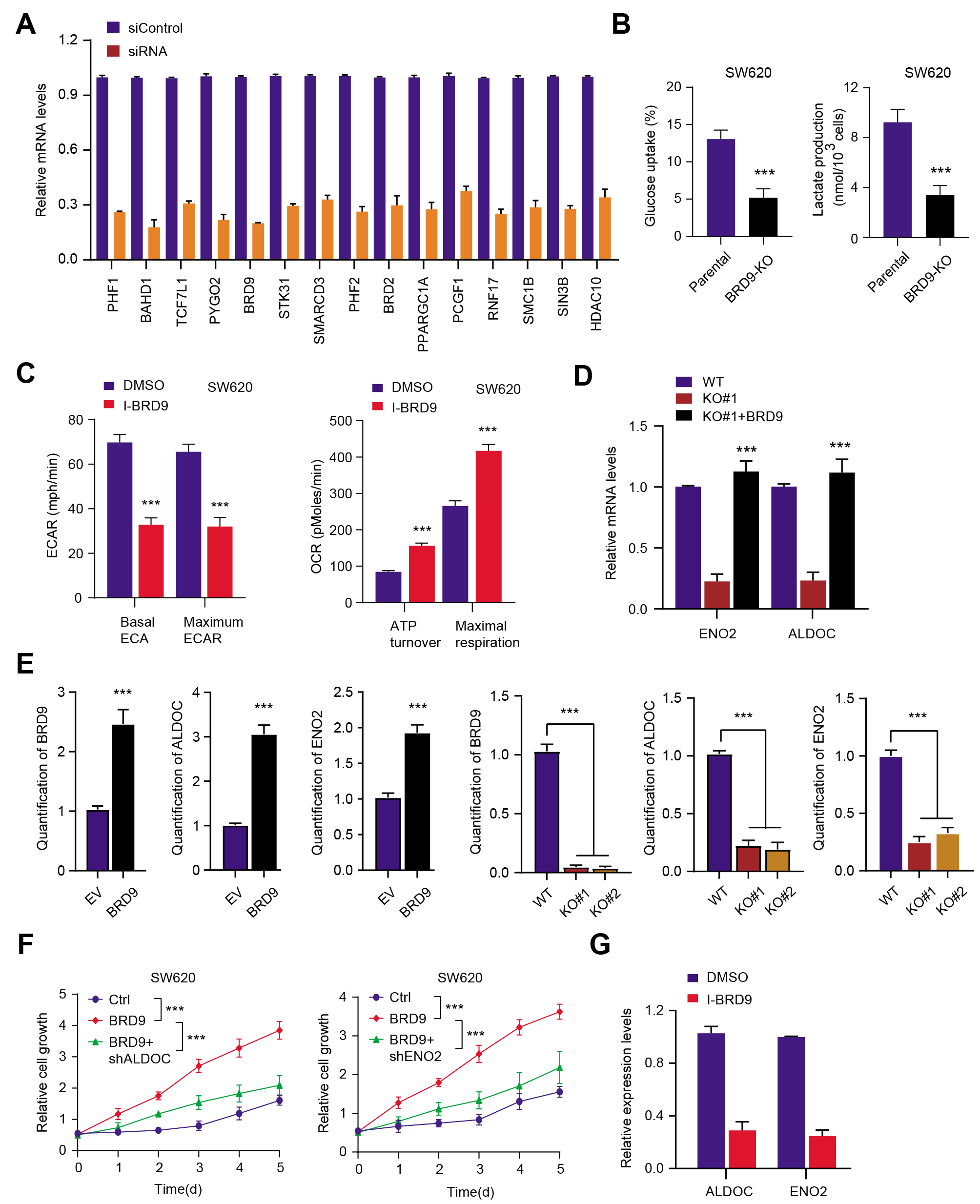

Supplement: Supplementary file 1 — Figure S1 [file CAM4-12-1572-s004.jpg]
